# Doctor Recommendation Model Based on Ontology Characteristics and Disease Text Mining Perspective

**DOI:** 10.1155/2021/7431199

**Published:** 2021-08-08

**Authors:** Chunhua Ju, Shuangzhu Zhang

**Affiliations:** ^1^Business Administration College, Zhejiang Gongshang University, Hangzhou, China; ^2^School of Management Science and Engineering, Zhejiang Gongshang University, Hangzhou, China

## Abstract

**Background:**

Patients can access medical services such as disease diagnosis online, medical treatment guidance, and medication guidance that are provided by doctors from all over the country at home. Due to the complexity of scenarios applying medical services online and the necessity of professionalism of knowledge, the traditional recommendation methods in the medical field are confronting with problems such as low computational efficiency and poor effectiveness. At the same time, patients consulting online come from all sides, and most of them suffer from nonacute or malignant diseases, and hence, there may be offline medical treatment. Therefore, this paper proposes an online prediagnosis doctor recommendation model by integrating ontology characteristics and disease text. Particularly, this recommendation model takes full consideration of geographical location of patients.

**Objective:**

The recommendation model takes the real consultation data from online as the research object, fully testifying its effectiveness. Specifically, this model would make recommendation to patients on department and doctors based on patients' information of symptoms, diagnosis, and geographical location, as well as doctor's specialty and their department.

**Methods:**

Utilizing crawler technique, five hospital departments were selected from the online medical service platform. The names of the departments were in accordance with the standardized department names used in real hospitals (e.g., endocrinology, dermatology, gynemetrics, pediatrics, and neurology). As a result, a dataset consisting of 20000 consultation questions by patients was built. Through the application of Python and MySQL algorithms, replacing semantic dictionary retrieval or word frequency statistics, word vectors were utilized to measure similarity between patients' prediagnosis and doctors' specialty, forming a recommendation framework on medical departments or doctors based on the above-obtained sentence similarity measurement and providing recommendation advices on intentional departments and doctors.

**Results:**

In the online medical field, compared with the traditional recommendation method, the model proposed in the paper is of higher recommendation accuracy and feasibility in terms of department and doctor recommendation effectiveness.

**Conclusions:**

The proposed online prediagnosis doctor recommendation model integrates ontology characteristics and disease text mining. The model gives a relatively more accurate recommendation advice based on ontology characteristics such as patients' description texts and doctors' specialties. Furthermore, the model also gives full consideration on patients' location factors. As a result, the proposed online prediagnosis doctor recommendation model would improve patients' online consultation experience and offline treatment convenience, enriching the value of online prediagnosis data.

## 1. Introduction

As the emphasis of medical care gradually shifts from disease to patient, the role of patients' participation in online health improvement is becoming more prominent. The health service in the world is not only different in terms of regions but also varying in terms of online health services [1, 2]. Specifically, there exist phenomenon such as information asymmetry between doctors and patients and unequal distribution of medical resources geographically [3]. Therefore, patients registering doctors online and intelligent department recommendation have also become one of the important topics of medical informatization. According to a report released in 2019 by the Big Data Research Institute, the scale of users in China's medical and health market was about 800 million by the end of 2018 [4]. With a large number of doctors and patients interacting online, a large amount of real consultation data has been accumulated in the online health community. Therefore, it is of important theoretical and practical value to investigate how to make full use of online data to build models to improve patients' medical treatment experience in terms of increasing the accuracy of patients' medical choice and the effectiveness of department recommendation.

The existing literature has been conducting studies from perspectives of department recommendation and doctor recommendation. The two methods of department recommendation are separately based on expert system and similarity calculation. As for department recommendation based on the expert system, on one hand, through establishment of medical knowledge base with the help from medical experts, the diagnosis process of medical experts is simulated by applying rule-based reasoning engine. As a result, patients' diseases are predicted, so as to achieve the target department recommendation for patients. Moreover, the expert-based department recommendation is built upon fuzzy logic and RBF neural network, effectively improving the recommendation accuracy [5, 6]. On the other hand, there exist many problems due to the abundant number of reasoning rules, such as low computational efficiency and high maintenance cost of knowledge base. As for department recommendation based on similarity calculation, the current literature uses various methods to measure similarities, such as similarity between patients' symptoms and disease' symptoms [7], TF-IDF sentence-based similarity and TF-IDF algorithm that is based on multiple words [8, 9], combination of focus shifting backwards, and professional medical corpus [10]. This similarity-based recommendation would, respectively, calculate the possibility of having disease and descriptive words that may correspond with certain symptoms, realizing the goal of department recommendation to patients. Research of recommendation on doctor is mainly based on the content and collaborative filtering recommendation algorithm, focusing on user keywords, browsing history, evaluation, and other data [11, 12]. The user collaborative filtering algorithm assumes that one user and other user group who share similar interest would have same product preference [13–15]. Among them, user collaborative filtering algorithm integrating projects mainly solves the problem of information overload through filtering attribute collaboratively [16]. Moreover, the application of customized relational network and tags solves the problem of data sparsity in the matrix factorization recommendation model [17, 18], and the collaborative filtering recommendation method integrates contextual perception, project similarity, and user behavior, giving recommendation results from perspectives of patients' contexts, projects, and user participation [19–21]. In addition, scholars also conducted modeling research on doctor recommendation, disease diagnosis, and medical examination [22, 23] from the perspectives of semantic characteristics of medical resources [24], user information types [25], user ratings, and comment portraits [26], as well as Bayesian algorithm [27].

The recommendation algorithms in the traditional medical field mainly have the following three problems. First, in terms of department recommendation, the algorithm based on the expert system causes problems such as explosion of knowledge rule reasoning and high maintenance cost of knowledge base. Furthermore, the algorithm based on similarity may not effectively recognize synonyms, possibly decreasing recommendation accuracy. Second, in terms of doctor recommendation, the user-based collaborative filtering algorithm may cause problems that patients of similar symptoms would not be diagnosed with the same disease, due to complexity and diversity of diseases. What is more, because of the nonnecessary relationship among patients' etiologies, the assumption of the project-based collaborative filtering algorithm that users would choose doctors with the same research field as their previous doctors may hardly be met. Third, although relevant literatures have studied how to reduce data sparsity [28–30], the collaborative filtering recommendation algorithm still cannot completely avoid the performance problems caused by data sparsity.

Based on the above theorization, it can be concluded that the existing recommendation algorithms cannot fully meet requirements with regard to recommendation in the context of the Internet medical field. Patients can access medical services provided by doctors in the online health community all over the country online without going out, including disease diagnosis, medical treatment guidance, and medication guidance. Meanwhile, patients consulting online come from far and near and may involve situations of offline medical treatment, making it necessary to take into account the factor of patients' location. Therefore, this paper proposes an online prediagnosis doctor recommendation model that integrates ontology characteristics and disease text mining, improving both the effectiveness of doctor recommendation within the environment of online medical service and the convenience of offline medical treatment for patients.

## 2. Research on the Doctor Recommendation Model

**Figure 1:**
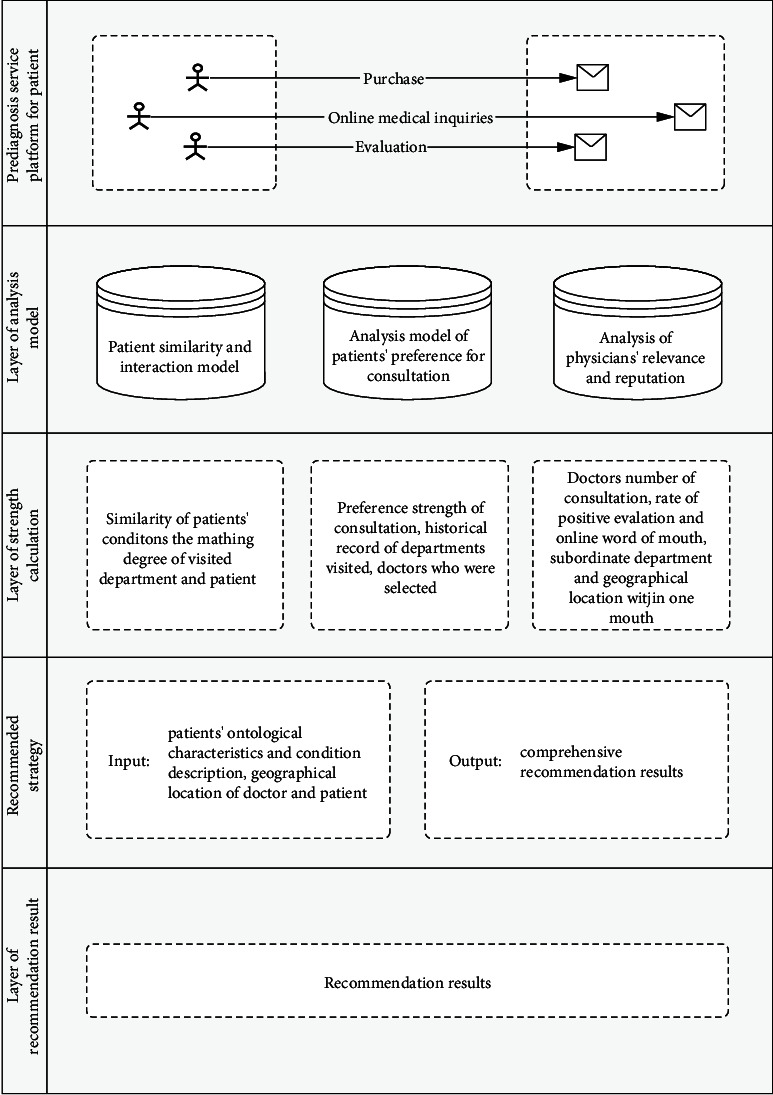
Prediagnosis doctor recommendation model integrating ontology characteristics and disease text mining.

The doctor recommendation model is mainly divided into three steps. Step 1: data preprocessing. Perform word segmentation and stop word removal with regard to patient's input of natural language. Step 2: hospital department recommendation. After screening patients' query data, create the most similar sentence set based on key parts of word vector or the similarity measurement for symptom descriptions, so as to achieve department recommendation. Step 3: doctor recommendation. Use SQL sentence query in the MYSQL database to complete doctor recommendation (Figure 1).

## 3. Data Cleaning Process

**Figure 2:**
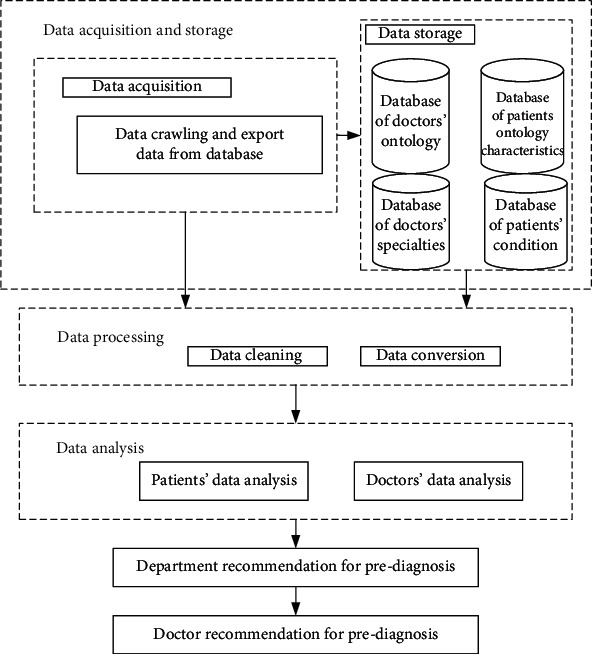
Data cleaning process.

There are mainly two aspects of data that are available online. The first aspect of data is patients' online consultation regarding disease symptom. This source of information mainly covers age, gender, symptom description, and other data. The second aspect of data is doctors' information online, including doctors' names, titles, hospitals, departments, and their specialties as shown in Table 1. All data is in structured form, and information such as disease description, prediagnosis, and specialties are stored in text form. Then, model will be built after word segmentation and keyword extraction (Figure 2).

## 4. Data on Ontology Characteristics of Doctors and Patients

The doctor-patient demographic data obtained from WeiYi platform are mostly well-organized semistructured textual data. The first step is to transform unstructured text data into structured text data through named entity recognition and information extraction. Organization names, people's names, and location names can be recognized by applying multiple open source Chinese language processing tools [31], such as fudanNLP developed by Fudan University [32], NLPIR word segmentation system developed by Chinese Academy of Sciences [33], and LTP Chinese natural language processing platform of Harbin Institute of Technology [34]. In addition, delete the missing value and duplicated information. And, for the problem of different doctors sharing one same name, use fields such as “the hospital to which they belong” and “the department to which they belong” to restrict.

## 5. Data on Patients' Condition Description

Data on patients' online condition description are presented as specific evaluations expressed by patients in natural language. The data in its initial form are fulfilled with problems that the contents are nonstandardized, repetitive, short, and single [35]. The authors marked the text content by part of speech and synonyms and then use human tissue lexicon and human anatomy lexicon to match the word segmentation results so as to extract disease symptoms and keywords of human body parts. As shown in Table 1, the patient's main complaint was that “it was caused by pelvic effusion eight years ago, there was no abortion history and no pregnancy.” The common clinical symptoms that the patient did not actually have appeared in the description make it difficult to extract keywords. For example, “no abortion history “ was divided into “no” and “abortion history,” resulting in the extraction of “ abortion history “ as the keyword; yet, the patient did not have these symptoms. To deal with situations like the abovementioned, before word segmentation, the authors would divide the description paragraph into short sentences or phrases by punctuation marks, and the stop words should be retained in word segmentation. Then, while extracting keyword, the target words cannot be considered as the real target keywords if they contain negative modifiers such as none, unaccompanied, and no.

## 6. Data on Doctors' Specialties

Data on doctors' specialties are structured textual data and are confronted with problems of synonymous naming and missing data. An example of synonymous naming refers to the problem that doctors in different hospitals have different naming for their fields of expertise. Specifically, synonyms for fields of expertise are specialties, being good at, specializing in, being skilled in, being professional with, medical interest, and research direction. All synonymous naming shall be integrated into the same field. As for the problem of missing data, utilize multiple data source data integration to complete improvement or deletion.

## 7. Doctor Recommendation

### 7.1. Department Recommendation

For questions input by patients, every keyword for each sentence can be obtained after word segmentation and word stopping removal. Next, the corresponding question set can be obtained by positioning question sentences that are associated with each keyword. The authors divided the question set into sample dataset and test dataset, both containing information of patients' condition description text, online prediagnosis department recommendation, etc. Then, use the word2vec library to train a word vector model on the keywords of the sentences in the sample data set, calculate the similarity between the questions input by the patient in the test data set and the word vector model of the sample data set, and lastly select the most similar questions to the sample data set in the test dataset. Following the rule that higher similarity indicates the same one department, after screening the similarity calculation one by one, the department with the highest similarity would be the final recommendation result.

## 8. Doctor Recommendation

The core significance of the development of online medical and health services is to reshape the medical service process and optimize the allocation of medical resources, so as to meet the medical and health needs of individual consumers. Due to its mobility, convenience, rapidness, personalization, and interaction, the online medical services have become the main channel for consumers to seek medical help online, having been adopted and utilized by consumers. To some extent, it alleviates the medical pressure and realizes the optimal allocation of medical resources. The patients using online medical service come from all sides, and the majority of them have conventional and chronic diseases, making it sometimes necessary for patients to confirm their diagnosis offline. Therefore, doctor recommendation that takes into account of patients' location information is particularly important to improve patients' convenience of offline medical treatment and to attract more patients to use online medical services. Based on the SQL statements query function in the MYSQL database, matching keywords with doctors' specialties, department, and region information, integrating patients' location information, and this paper recommends local doctors that meet the requirements according to patients' region. For instance, a patient's naming Zhang San, living in Zhejiang province, with condition described as thick endometrium, heavy menstrual flow, and stomachache, would be recommended to see a Chief Physician from Department of Gynecology at Zheyi hospital with family name of Wang.

## 9. Sentence Similarity

### 9.1. Calculation of Similarity Based on Postcontent

After obtaining the unique *d*-dimensional distribution vector representation of the disease description text content, the similarity and distance between each two text contents can be obtained through similarity calculation. The author uses the cosine formula to measure the similarity between two texts and uses the Mahala Nobis distance to calculate the natural language description of the two posts. Assume that two paragraph vectors of natural language description of text content are expressed as PV_*a*_ = (×11, ×12, ⋯, ×1*d*) and PV_*b*_ = (×21, ×22, ⋯, ×2*d*), where *d* represents two paragraph vectors. The similarity and distance are defined as follows:

(1) $$\begin{array}{c} \begin{array}{c} \text{sim}\left(\text{PV}a,\text{PV}b\right)=\frac{\text{PV}d•\text{PV}d}{{\left\|\text{PV}d\right\|}^{2}•{\left\|\text{PV}d\right\|}^{2}},\\ =\frac{\sum_{i-0}^{i=d}x1ix2i}{\sqrt{\sum_{i-0}^{i=d}x_{1i}^{2}\sqrt{\sum_{i-0}^{i=d}x_{2i}^{2}}}}, \end{array}\\ \text{dis}\left(\text{PV}a,\text{PV}b\right)=\sqrt{{\left(\text{PV}a-{\text{PV}}_{b}\right)}^{T}S^{-1}\left(\text{PV}a-\text{PV}b\right)}, \end{array}$$

where *S* is the covariance matrix of eigenvectors PV*_a_* and PV*_b_*.

### 9.2. TF-IDF Sentence Similarity Based on Co-Occurring Words

This method believes that in two sentences, the more the same vocabulary, the higher the similarity of the two sentences ^[36]^. Specifically,

(2) $$\begin{array}{c} \text{SimScore}\left(S1,S2\right)=\frac{\left|S1\cap S2\right|}{\left|S1\cup S2\right|}\underset{wi\in S1\cap S2}{\sum }\text{w}\text{eight}\left(wi\right),\\ \text{weight}\left(wi\right)=\frac{\text{Num}\left(wi,k\right)}{Nk}\times \log\left(\frac{Nt}{\text{Num}\left(wi,t\right)+1}\right). \end{array}$$

Among them, |·| is the cardinality of the set, *S*_1_ and *S*_2_ are the word sets of the two sentences to be compared, *w*_*i*_ represents the symptom word *i* in the department question and answer sentence, weight (*w*_*i*_) is the TF-IDF ^[37]^ weight, Num (*w*_*i*_,*k*) represents the number of sentences in which the symptom word *w*_i_ appears in the question and answer sentence set of department *k*, *N*_*k*_ represents the number of all questions and answers in department *k*, *N*_t_ represents the total number of questions and answers in the knowledge base, and Num (wi, *t*) represents the total number of questions and answers in the knowledge base. The number of sentences in which the symptom word *i* appears in the question. The TF-IDF sentence similarity calculation method based on co-occurring words belongs to the surface structure analysis method. It simply uses the surface information of the sentence, that is, the word frequency, part of speech, and other information of the words in the sentence to calculate the sentence similarity, without considering synonyms. This results in a decrease in the accuracy of sentence similarity.

### 9.3. Sentence Similarity Method Based on Word Vector

Word vector sentence similarity is mainly used indepth learning tool word2vec ^[38]^ to process words into vectors and obtain the semantic similarity of sentence pairs to be compared by calculating the similarity between vectors. The specific formula is as follows:

(3) $$\begin{array}{c} \underset{wi\in I,wj\in R}{\text{CosSim}}\left(wi,wj\right)=\frac{\sum_{i=1}^{n}\left(xi,yi\right)}{\sqrt{\sum_{i=1}^{n}x_{i}^{2}}\times \sqrt{\sum_{i=1}^{n}y_{i}^{2}}},\\ \text{SimScore}\left(S1,S2\right)=\frac{\sum_{w\in \text{IR}}\beta wM\text{axSimValue}\left(\text{CosSim}\left(w,\text{IR}\right)\right)}{\sum_{w\in \text{IR}}\beta w}. \end{array}$$

Among them, IR = *S*_1_ ∪ *S*_2_, *w*_*i*_ and *w*_*j*_ are the two words to be compared, which represent the words in sentence *S*_1_ and the words in sentence *S*_2_, respectively; *n* represents the dimension of the word vector, and *x*_*i*_ and *y*_*i*_ represent the word vector of *w*_*i*_, and the vector value of the *i*th dimension of the word vector of *w*_*j*_; MaxSimValue (CosSim (*w*,·)) represents the maximum value of the cosine similarity between the word vector corresponding to word *w* and the word vector corresponding to all vocabulary of another sentence; parameter *βw* is The TF-IDF weight value of word *w* in the sentence. The greater the value of SimScore (S1, S2), the greater the similarity between the two sentences and the closer the semantics.

## 10. Experiment

### 10.1. The Data Set

To analyze the doctor recommendation method proposed in this paper, an experimental study was conducted. The data of five most common departments were crawled from the well-known domestic medical online platform-WeiYi. The names of the departments were in accordance with the standardized department names used in real hospitals (e.g., endocrinology, dermatology, gynemetrics, pediatrics, and neurology). As a result, a dataset with name of T consisting of 20000 patients' preclinical data online were built. To conduct experimentally comparative analysis of various algorithms, two widely used evaluation indexes for the recommendation performance were adopted in this paper, being accuracy rate (being *P*) and recall rate (being *R*):

(4) $$\begin{array}{c} P=\frac{\text{TP}}{\text{TP}+\text{FP}}R=\frac{\text{TP}}{\text{TP}+\text{FN}} \end{array}$$

### 10.2. Parameter Setting

In the experiment, the dimension parameter of the word vector was set as 100. With regard to the calculated similarity results of keyword set that would be used for department recommendation, take the top 5 questions with the highest sentence similarity as the recommended result data (top *N* = top 5), and the threshold value of keyword set similarity was set as 0.8; that is, when keyword and test set data were used for keyword similarity calculation, the result must exceed 0.8 to be included in the hospital department recommendation set. If there were 2 or more recommended hospital departments, it would be considered as no recommendation, being a special case.

## 11. Results and Analysis

Among the 20000 patients surveyed, 16170 were female (77.3%). This may be because women are often required to care of family health and other responsibilities in addition to work; also, women tend to pay more attention to health information than men. A total of 16800/20000patients (84.0%) were 30 to 45years of age. Because of old men with limited experiences in consulting physicians and obtaining medicines and children that cannot master online counseling skills, so, old men and children may not frequently consult physicians on the internet or ask their family members to perform online inquiries. In the 20000 records, 12600 of the physicians (63.0%) are chief physicians or associate chief physicians, while19400 hospitals (97.0%) were ranked 3A (see Table 2).In order to verify the feasibility and effectiveness of the proposed recommendation algorithms for department and doctor, the experiment was conducted to compare them with the content-based recommendation algorithm and user-based collaborative filtering algorithm. First, randomly extract 100 pieces of data from the dataset *T* based on the hospital department name and then perform word vector training. After the process of word segmentation and stop word removal for data of different departments, the keyword set was obtained, and the word vector model was trained using this keyword set (see Table 3). The word vector model consisted of patients' real consultation questions, and the other words excluding those questions within the group were considered as noise words, representing meaningless words unrelated to patient's consultation. Three different algorithms were all used to measure similarity for keywords to give hospital department recommendation (see results of three algorithms in Table 4).

Seen from Table 4, the proposed similarity recommendation method in this paper that incorporates ontology features and disease text data mining was the best when applied to consultation about selecting appropriate hospital department since the accuracy rate and recall rate were much higher than the other two algorithms. This is because the word vector sentence similarity measurement strategy can better measure the semantic similarity of sentences. For example, for sentence pairs “I went to the hospital to see the dentist and went home, dizzy, heavy head, runny nose” and “When I came back from the dentist, I started to feel Dizziness with symptoms of heavy head and runny nose”. If a co-occurring word-based measurement method based on co-occurrence words is used, the similarity value is low, because the sentence pair contains such things as (dizziness, dizziness), (heavy head, sinking head), and (runny nose, runny nose). Synonym pairs such as clear nose) make the content-based method relatively good, and the word vector method has the best effect, indicating that it can more accurately capture the underlying semantics of the sentence. On one hand, this is because the method in this paper can measure the similarity of keywords better. For instance, keywords of “headache, palpitation, insomnia” and keywords of “head distension and restlessness” were considered as similar. The results were better than the sentence similarity measurement based on collocates. On the other hand, the proposed method in this paper took fully consideration of factors such as location information of doctors and patients, as well as doctors' expertise field, which would not be the case for the content-based recommendation method that only takes the patient's disease information into account.

**Figure 3:**
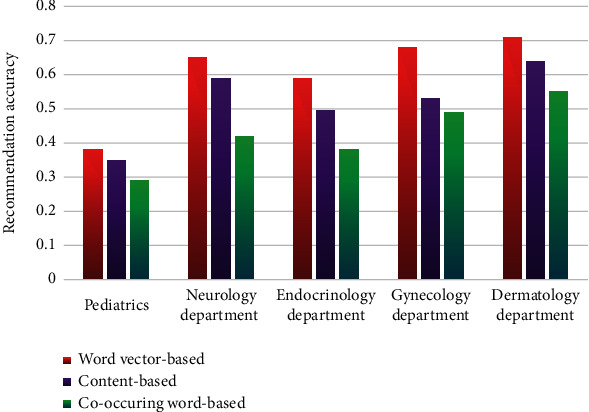
Recommendation accuracy comparison of different departments.

**Figure 4:**
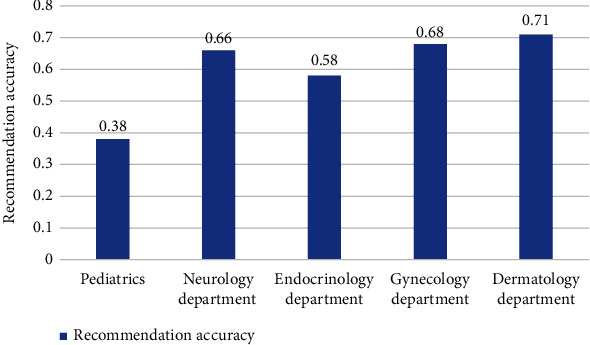
Comparison of recommendation rates of various departments.

Seen from Figures 3 and 4, the recommendation performance of the word vector method was varying for different hospital departments. The recommendation accuracy of pediatric department was below 0.5, and that of neurology, endocrinology, gynecology, and dermatology departments were all above 0.5, among which the recommendation accuracy of gynecology was the most improved. With regard to the four departments with relatively higher recommendation accuracy, including neurology, obstetrics, gynecology, and dermatology, what they had in common was that the characteristics of the consultation questions were very typical and obvious. For example, high blood sugar, sudden weight loss, and thirst are typical for endocrinology; red rash, circular rash, redness, swelling, and itching are typical for dermatology; pregnancy and irregular menstruation are typical for gynecology. However, the situation is different for pediatric department in that if information indicating age such as baby, child, and 6 months old is not included in the consultation, it may lead to the systematic recommendation to other departments, reducing the accuracy accordingly.

**Figure 5:**
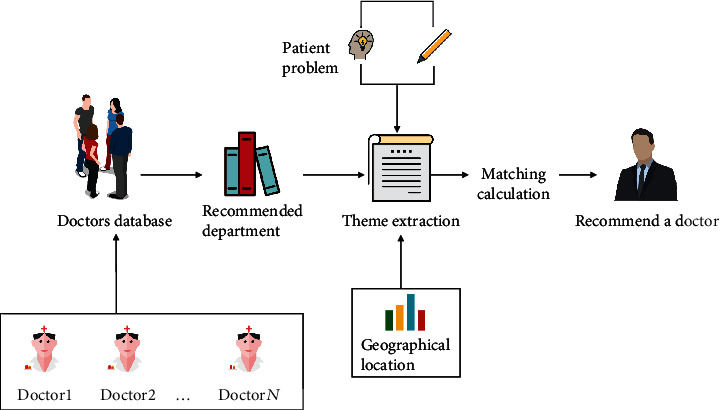
Doctor recommendation framework.

Finally,The SQL statement query function in the MYSQL database used to integrate the patient's regional factors. According to the patient's region, we use the department and regional keyword matching and recommend the doctors in the hospital to patient in the region that meet the needs, such as “Zhang San, from Zhejiang, the condition is described as uterus Thick intima, heavy menstrual flow, and stomachache,” and the recommended doctor is “Zhejiang First Hospital-Gynecology-Dr. Wang (Chief Physician).” The process is shown in Figure 5.

## 12. Conclusion

Traditional manual medical guidance is increasingly unable to meet the people's medical needs, registration is difficult, and the problem of not finding a clinic has become increasingly prominent. Aiming at the shortcomings of traditional medical department recommendation research methods and factors such as the necessity for professional medical diagnosis expertise and information asymmetry between doctors and patients makes it impossible for patients to identify the appropriate clinic room or doctors. Once mistakes are made, online consultation time would be wasted, increasing the cost of hospitals and patients when the patient goes offline instead for medical treatment. In this paper, the proposed online prediagnosis doctor recommendation model integrates ontology characteristics and disease text mining. The experimental process uses real data on the Internet medical comprehensive website and is similar to the sentence based on content based, and based on collocate based is compared; the experiment verifies the reliability and effectiveness of the method in this paper. This provides great convenience for patients to seek medical treatment and at the same time reduces medical costs. It gives a relatively more accurate recommendation advice based on ontology characteristics such as patients' description texts and doctors' specialties. As a result, the proposed online prediagnosis doctor recommendation model improves patients' online consultation experience and offline treatment convenience, enriching the value of online prediagnosis data. In addition, the primary real data from the online medical consultation platform were utilized to verify the reliability and effectiveness of the proposed method.

## 13. Limitations

It is not without limitation in this paper. First of all, this study was only carried out based on data from one online medical community, rendering its generalizability a question. Future study may consider collecting data from multiple online medical community platforms to verify the recommendation effect of the proposed algorithm. Second, considering that this study is solely focused on the proposed recommendation model for Chinese patients, similar studies shall be carried out in Western background in the future. Third, because of the complexity of the medical domain knowledge, follow-up researches shall not only incorporate techniques such as semantic analysis and sentiment analysis to expand the sample into general practice data but also consider introducing users' other behavioral information to introduce the user information behavior factor optimize the target object, for intelligent department recommendation tasks, in addition to controlling data quality and deep learning algorithms such as LSTM shall be applied to improve model accuracy in the future. The intelligent department recommendation task can also be abstracted as a multilabel classification task for texts. Accordingly, multiple department categories can be recommended for patients' questions covering multiple departments, etc. to further improve the accuracy of the proposed recommendation model, expecting to apply it to more online medical consultation platforms.

## Figures and Tables

**Algorithm 1:**
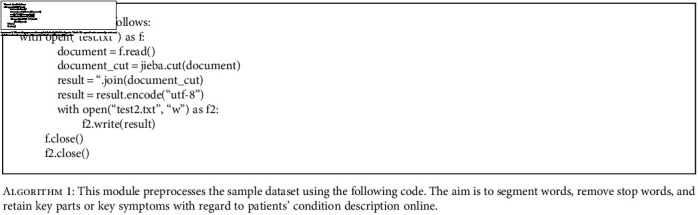
This module preprocesses the sample dataset using the following code. The aim is to segment words, remove stop words, and retain key parts or key symptoms with regard to patients' condition description online.

**Algorithm 2:**
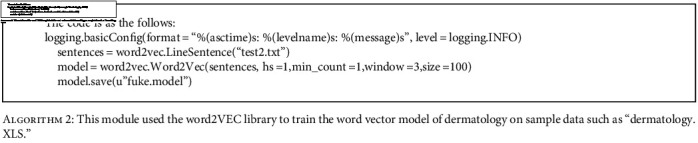
This module used the word2VEC library to train the word vector model of dermatology on sample data such as “dermatology. XLS.”

**Algorithm 3:**
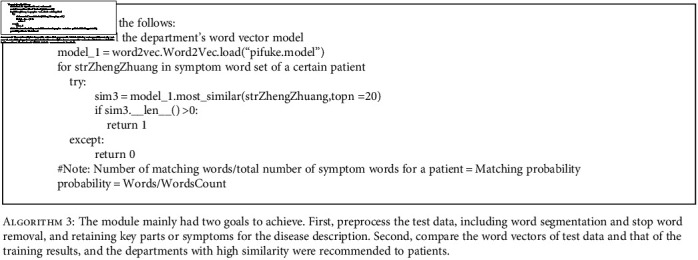
The module mainly had two goals to achieve. First, preprocess the test data, including word segmentation and stop word removal, and retaining key parts or symptoms for the disease description. Second, compare the word vectors of test data and that of the training results, and the departments with high similarity were recommended to patients.

**Table 1:** Data sample on patients and doctors online.

Patient ID	Gender	Age	Province/city	Main complaint	Initial consultation department online
8070844	Female	65	Jiangsu	Menstruation keeps coming. B-ultrasound result shows that my endometrium is thick. I ate progesterone and did curettage. For now, I have been taking medicines for 10 days. 3 days after progesterone, I still had large amount of blood flow, and my stomach ached. I am wondering what is wrong with me.	Gynecology
81305510	Female	42	Guangdong	Bilateral hydrosalpinx. I never had abortion history. I want to be pregnant now, what should I do now?	Gynecology
12031251	Female	43	Heilongjiang	43-year-old, irregular menstruation for many years, 3 times for 2 months, the period was long for 7/8 days, the amount is little, and the color is dark brown. What medicine should I take?	Gynecology
57715499	Female	37	Henan	Just had miscarriage a month ago; yet, I got pregnant in confinement. Can I keep the child?	Gynecology
72520784	Female	53	Shanghai	My mother is 53 years old. She feels nervous, unable to breathe, cannot lie down, and feels no strength.	Neurology
Doctor name	Title	Hospital	City	Specialties	Department
Niu^∗∗^	Chief Physician	Ningbo First Hospital	Ningbo	Diagnosis and treatment of diabetes and thyroid disease	Endocrinology
Yang^∗^	Associate Chief Physician	Shijiazhuang First Hospital	Shijiazhuang	Hemorrhagic cerebrovascular disease such as cerebral aneurysm, arteriovenous malformation, arteriovenous fistula, and cavernous hemangioma; ischemic cerebrovascular diseases such as carotid artery stenosis, vertebral artery stenosis, intracranial artery stenosis,and moyamoya disease	Neurosurgery
Xu^∗∗^	Chief Physician	Beijing Anzhen Hospital, Capital Medical University	Beijing	Diagnosis, surgical treatment, and perioperative treatment of various congenital heart diseases	Pediatric cardiac surgery
Wang^∗∗^	Associate Chief Physician	Shenzhen Bao'an People's Hospital	Shenzhen	Diagnosis and treatment of diabetes and its complications, hyperthyroidism, and hypothyroidism; use of insulin pump and dynamic blood glucose monitors	Endocrinology
Liu^∗∗^	Chief Physician	Hospital of Traditional Chinese Medicine in Uygur, Xinjiang	Xinjiang	Neurology of traditional Chinese medicine	Neurology

^∗^ and ^∗∗^ mean one word or two words for the Chinese name.

**Table 2:** Summary of the characteristics of the collected data records (*N* = 20000).

Characteristic	Value, *n* (%)
Gender	
Male	4540 (33.7)
Female	15460 (77.3)
Age (years)	
25-30	1586 (7.9)
31-45	16800 (84.0)
46-50	1014 (5.1)
>55	600 (3.0)
Physician's professional title	
Resident physician	2670 (13.35)
Attending physician	4330 (21.65)
Associate chief physician	8040 (40.2)
Chief physician	4560 (22.8)
Other	400 (2.0)
Hospital's ranking level	
3A	19400 (97.0)
Other	600 (3.0)

**Table 3:** Word vector model and keyword examples.

Word vector-based model	Keyword set	Department
	Headache, nausea, right eye, swelling, stuffy nose, right ear, tinnitus, etc.	Neurology
Keyword set	1. Migraines, nausea, loss of appetite 2. Headache, dizziness, protrusion of left eye, congestion of eyeball 3. Head distension, stuffiness, dizziness, palpitation, and restlessness 4. Palpitations and palpitations 10. Weak right hand, unable to clench a fist, palpitation, unable to breathes	

**Table 4:** Comparison of accuracy and recall rate.

Algorithm method	Accuracy rate (%)	Recall rate (%)
Word vector-based	74	78
Content-based	63	67
Co-occurring word-based	54	56

## Data Availability

The data were collected with help from the administrator of the WeiYi platform. Due to third-party rights, patient privacy, and commercial confidentiality, data is not open source.
